# Signaling Molecules and Diagnosis of Cognitive Disorders: Current State and Prospects

**DOI:** 10.3390/ijms27010372

**Published:** 2025-12-29

**Authors:** Igor Kvetnoy, Oleg Kheyfets, Lazar Safaniev, Vladimir Kheifets, Ekaterina Mironova, Tatiana Kvetnaia, Gianluigi Mazzoccoli, Kiryl Prashchayeu, Anna Gavrilova

**Affiliations:** 1 Medical Institute, Saint-Petersburg State University, 199034 Saint-Petersburg, Russia; igor.kvetnoy@yandex.ru; 2 Saint-Petersburg Research Institute of Phthisiopulmonology, 191036 Saint-Petersburg, Russia; 3 University Hospital Wiener Neustadt, 2700 Wiener Neustadt, Austria; post@urologie-kheyfets.at; 4 Department of Medicine, Danube Private University, 3500 Krems an der Donau, Austria; 5 Medical Research Company “A-Biomarker”, 125080 Moscow, Russia; 6 Almazov National Medical Research Center, 197341 Saint-Petersburg, Russia; 7 Saint-Petersburg Institute of Bioregulation and Gerontology, 197110 Saint-Petersburg, Russia; 8 Chronobiology Laboratory, Foundation IRCCS Casa Sollievo della Sofferenza, Opera di Padre Pio da Pietrelcina, 71013 San Giovanni Rotondo, Italy; 9 Medical Research Center «Gerontology», 129226 Moscow, Russia

**Keywords:** Alzheimer’s disease, dementia, biomarkers, differential diagnosis, buccal epithelium

## Abstract

Cognitive disorders present significant medical and social challenges nowadays, due to their high prevalence, progressive course and a lack of effective methods for treatment of neurodegenerative diseases and comorbid pathologies. An important area of research is the identification of molecular biomarkers that reflect early pathophysiological changes and facilitate a more accurate biological characterization of cognitive impairment. This study provides an overview of the most relevant signaling molecules for diagnosing cognitive disorders. It presents data on the effectiveness of using comprehensive panels of molecular biomarkers in clinical practice, including β-amyloid, CD34, claudin, DRP1, endothelin-1, NF-kB, PINK1, RAGE, S100, α-synuclein, and tau protein, in patients with Alzheimer’s disease (AD) and vascular dementia (VD). The study results demonstrate that cumulative changes in the expression of signaling molecules reflect various neurodegenerative and vascular-associated biological processes. The data obtained are comparative in nature and require further validation before potential clinical application.

## 1. Introduction

Cognitive impairments primarily arise due to vascular risk factors and various forms of cerebrovascular pathology. According to recent data, vascular cognitive disorders account for at least 20–40% of all cases of dementia [1]. According to the World Health Organization, approximately 55 million people lived with dementia in 2019, and this number is expected to increase to approximately 139 million by 2050 [2]. According to the Global Burden of Disease study (GBD, 2019), Alzheimer’s disease (AD) and other dementias accounted for approximately 25.3 million disability-adjusted life years (DALYs) lost [3,4,5]. From 1990 to 2019, the number of DALYs attributable to AD and other dementias increased from ~9.7 million to ~25.3 million, reflecting the impact of population aging and improved life expectancy in most regions of the world [6]. In 2018, the number of people with dementia in the world was estimated to be around 50 million, and it is expected to triple by 2050. The prevalence of vascular cognitive impairments varies by country income levels: in low- and middle-income countries, the figures are still lower, but it is these countries that show the fastest growth in incidence. Hypertension, atherosclerosis, and other vascular risk factors are extremely common among elderly people, which is confirmed by numerous research data [7]. All together, this explains an increase in the number of strokes, dementia, and their combined forms: approximately one in three people over 65 experiences at least one of these conditions.

This term (cognitive impairment) refers to a broad spectrum of dysfunctions, from mild cognitive decline to severe dementia [8]. These conditions may be caused by ischemic and hemorrhagic strokes, chronic exposure to vascular factors, and their combination with neurodegenerative processes, including AD [9].

Vascular cognitive impairments are caused by a variety of pathological processes: small vessel disease, atherosclerosis of large arteries, cerebral hemorrhages, and pulmonary embolism. Such changes lead to impaired cerebral blood flow, hypoxia, dysfunction of the blood–brain barrier, inflammatory reactions and, as a consequence, the development of vascular dementia (VD) [10]. Dysfunction of this system is viewed as a key mechanism contributing to the progression of cognitive deficit. A chronic reduction in cerebral blood flow may cause atrophy, white matter damage, lacunar strokes, microbleeds, which in turn are associated with deterioration of memory, attention and executive functions [11]. The accumulation of ischemic lesions, even in asymptomatic cases, significantly increases the risk of developing dementia.

A chronic, age-associated dysregulation of cerebral blood flow appears to be a key pathophysiological mechanism underlying most vascular disorders [12]. Also important are inflammatory processes, endothelial dysfunctions, and cardiovascular pathologies. Certain risk factors typical for middle age, such as arterial hypertension, hypercholesterolemia, diabetes and smoking, increase the probability of developing dementia by 20–40% [13]. The combined impact of several risk factors increases this figure even more. Control of vascular risk factors, including by means of complex multimodal approaches with mandatory lifestyle changes, is currently viewed as the most promising strategy for the prevention and treatment of cognitive disorders.

Neurodegenerative diseases remain one of the most significant medical and social problems, which is due to their high prevalence, progressive course and a lack of effective treatment methods. More and more scientific evidence confirms the leading role of cerebrovascular pathology not only as an independent cause of cognitive decline, but also as a factor aggravating the course of neurodegenerative diseases [14]. AD (senile dementia of the Alzheimer’s type) is a progressive neurodegenerative disorder characterized by the irreversible deterioration of cognitive and physical functions. AD is the most common form of dementia in the world: according to various data, this neurodegenerative disease currently affects 25 to 40 million elderly people worldwide. According to the World Health Organization (WHO), the prevalence of AD will increase three to four times by 2050 [15]. AD is characterized by progressive memory loss, severe dementia (mental disability) and may become, according to WHO experts, one of the most likely causes of death in the 21st century.

Lifetime diagnosis of AD is difficult, since various forms of senility are often found in other neurodegenerative diseases, which complicates the timely targeted optimal treatment [16]. Diagnosing a specific form of dementia as a manifestation of AD can be definitively confirmed only by a pathologist during an autopsy and brain examination using certain histochemical and immunohistochemical methods. The development of reliable methods for the in vivo identification of AD biomarkers in a laboratory will allow for a timely choice of an appropriate method for pathogenetically justified therapy aimed at improving the patients’ psychosomatic condition and social orientation, and will also create a foundation for developing effective methods for the prevention of severe neurodegenerative pathologies [10,11].

Given their high prevalence and serious socioeconomic consequences, cognitive impairments are becoming one of the most important problems in modern medicine. Nowadays, prevention—early detection and correction of vascular risk factors using multimodal interventions—is considered the most effective approach [17,18].

The American Heart Association has proposed unified criteria for describing vascular cognitive impairments, encompassing the entire spectrum—from mild cognitive dysfunction to dementia [19]. The search for biomarkers of VD and AD is fundamentally important for modern neuroscience and medicine in general. These diseases develop gradually, and their clinical manifestations become noticeable only when the pathological process has already gone too far. Biomarkers can indicate biological changes that precede overt clinical manifestations and contribute to a deeper understanding of disease mechanisms.

## 2. The Role of Biomarkers in Dementia Diagnosis: Accuracy, Early Identification, and New Opportunities

**Figure 1:**
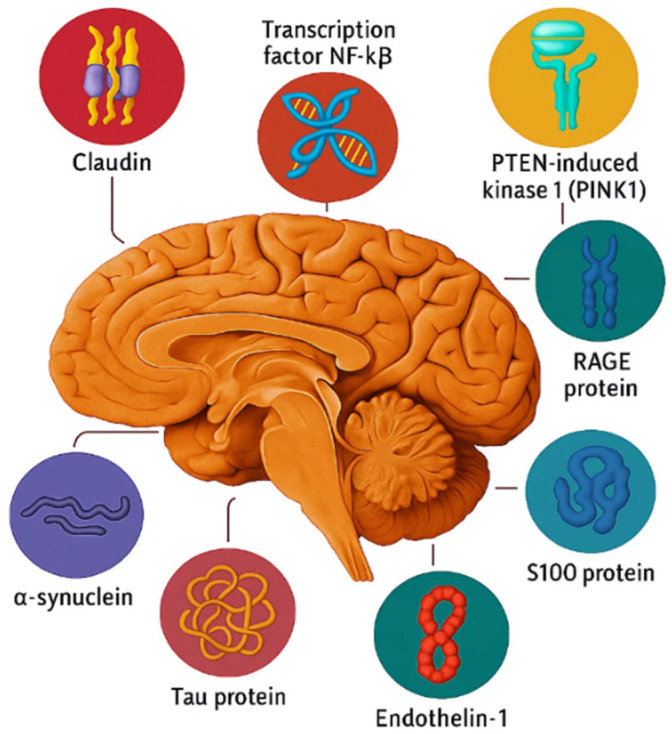
Biomakers of neurodegenerative diseases.

The particular value of biomarkers lies in their ability to differentiate between VD and AD, as the clinical symptoms of these diseases often overlap: impairments in memory, attention, and executive functions are observed in both conditions (Figure 1). However, these impairments differ in their nature: vascular dementia is characterized by ischemic lesions, white matter damage, microinfarctions, micro- and macrohemorrhages, and blood–brain barrier dysfunction. Currently, four molecules are considered to play a key role in the mechanism of AD onset and development: beta-amyloid, tau protein, ubiquitin, and acetylcholine. In its recommendations, the Alzheimer’s Disease Consortium, established by the National Institute on Aging and the Reagan Institute (both in the United States), points out that abnormal metabolism of these molecules is a key component of Alzheimer’s disease pathogenesis [20].

The use of biomarkers not only helps differentiate these pathologies but also predicts the rate of cognitive decline progression. In AD, important markers include levels of β-amyloid, total and phosphorylated tau protein, and neurofilament light protein, which reflects neuronal damage. Key indicators of vascular dementia include white matter hyperintensities on MRI, the presence of microinfarctions, micro- and macrohemorrhages, structural vascular changes, and vascular stiffness markers [1,21]. It is also important to note that a significant part of patients experience mixed dementia, where vascular and neurodegenerative mechanisms overlap, and in such cases, the combined use of biomarkers from both groups helps most accurately assess the nature of the pathology.

An equally important area is the use of biomarkers for monitoring treatment effectiveness. In AD clinical trials, they are used to track the dynamics of amyloid and tau protein deposition under the influence of new anti-amyloid medications [22]. In the case of vascular dementia, monitoring biomarkers allows for assessing the impact of antihypertensive and lipid-lowering therapy, as well as non-pharmacological interventions, including lifestyle changes, on cerebral circulation and cognitive function.

Finally, the integration of biomarker data with genetic risk factors, such as APOE4 allele status, and evaluating the patient’s vascular health can provide the basis for personalized medicine. This approach offers individualized preventive measures and therapeutic strategies, which are particularly important in view of the high prevalence of cognitive impairments in old age. The search for and validation of biomarkers for VD and AD is a prerequisite for progress in early diagnosis, disease differentiation, prognosis, and the development of effective and personalized treatment and prevention strategies [22].

In the context of AD, a major cause of dementia, searching for biomarkers is focused on key pathological processes: the accumulation of beta-amyloid (Aβ) and hyperphosphorylated tau protein. The amyloid hypothesis postulates that impaired Aβ catabolism, especially an elevated Aβ42/Aβ40 ratio, leads to the formation of neurotoxic oligomers and fibrils that form senile plaques, which trigger a pathological cascade of events [23,24].

### 2.1. β-Amyloid

The 40-amino acid extracellular protein is the most common form of β-amyloid (Aβ), accounting for up to 90% in the brain of healthy individuals and up to 40% in the brain of individuals with AD. However, mutations in the *APP* (amyloid precursor protein) gene promote the accumulation of Aβ with 42 amino acid residues (Aβ42), which is the most aggregation-prone form. The Aβ42 accumulation leads to the formation of amyloid oligomers, which, in turn, also aggregate and participate in the formation of senile plaques [25].

It has been found that Aβ42, which is considered the most toxic amyloid, predominates in brain tissues in patients with AD. Initially, aggregating Aβ was believed to form senile plaques in the brain, causing disruption of synaptic transmission, neuronal death, and, consequently, the development of dementia. Yet, the amyloid hypothesis could not explain the fact that the number and size of senile plaques in the brains of AD patients do not correlate with the degree of cognitive impairment. Moreover, some individuals without AD symptoms have extensive Aβ deposits, while some patients with inherited AD (IAD) have no senile plaques at all [12].

The degree of cognitive impairment in AD has been found to correlate with the biochemically verified amount of Aβ, but not with the histologically determined amount of Aβ. Soluble forms of Aβ remain invisible during immunohistochemical analysis. Probably, it is oligomers, rather than senile plaques, that are most toxic to neurons and cause pathological changes in AD patients.

Research findings in this area have led to a revision of the “amyloid hypothesis,” which has come to be called the “toxic oligomer hypothesis.” This new version of the amyloid hypothesis asserts that the accumulation of toxic Aβ42, aggregated in oligomeric assemblies, triggers a complex set of processes at the molecular and cellular levels, which ultimately leads to neuronal dysfunction and apoptosis.

Amyloid oligomers have been shown to impair the effect of long-term potentiation (LTP). LTP is a specific function of the nervous system that enhances the synaptic transmission between neurons and persists for a long time after the synapse is affected. LTP underlies synaptic plasticity, an ability of the nervous system to adapt to changing environmental conditions. It is widely believed that LTP plays a leading role in memory and learning processes.

Synaptic dysfunction, reduced LTP effects, and subsequent memory loss in early AD may be partially induced by amyloid oligomers, which disrupt synaptic remodeling and short-term memory formation.

Amyloid oligomers have been shown to cause oxidative stress and pathological changes in the endoplasmic reticulum, increasing the sensitivity of neurons to excitotoxicity—a pathological condition resulting in the suppression and death of neurons due to hyperactivation of glutamate receptors. Excitotoxicity is considered a cause of neuronal death in neurodegenerative diseases. Although this data supports the amyloid cascade hypothesis, its prevalence rate is around 1%. It should be noted, however, that the data supporting the amyloid cascade hypothesis were obtained in animal models of AD [26].

From a diagnostic perspective, recent advances in nanoscale imaging have significantly expanded the toolkit for detecting early Aβ aggregation. Atomic force microscopy (AFM) enables label-free imaging and nanomechanical mapping of amyloid fibrils and oligomeric structures in biological samples, complementing traditional biochemical methods [27,28]. Current reviews of AFM modes (high-resolution, force spectroscopy, nanochemical mapping) show that these approaches can reveal transient and nanoscale processes underlying neurodegeneration, including the formation of toxic Aβ oligomers [29,30]. Furthermore, AFM analysis of cerebrospinal fluid from patients with AD revealed specific fibrillar structures morphologically similar to amyloid and fibrin-like aggregates, which may reflect disruption of the blood–brain barrier and early amyloid processes, and are therefore considered promising ultra-early biomarkers of the onset and progression of AD [31]. Integration of AFM technologies with existing fluid biomarkers can significantly improve the accuracy of preclinical diagnostics.

### 2.2. Tau Protein

Tau protein (τ protein) is a protein associated with microtubules, which are essential for axon growth. Normally, τ protein interacts with tubulin, facilitating its assembly into microtubules and stabilizing their structure. Tau protein knockout mice have been found to develop progressive motor dysfunction with age [22].

Neurofibrillary pathologies associated with τ protein are found in the development of more than 20 neurodegenerative diseases. Hyperphosphorylated τ protein can spontaneously aggregate into paired helical filaments to form neurofibrillary tangles. In AD, hyperphosphorylated τ protein accumulates, which results in its dissociation from microtubules, destabilizing them, and leading to disruption of neuronal transport.

The number of neurofibrillary tangles correlates with the degree of disease progression but does not correspond to the degree of neuronal loss, as, according to experimental data, memory loss and neuronal death precede the formation of neurofibrillary tangles [26].

Some authors believe that it is τ-protein oligomers, rather than senile plaques, that exert a cytotoxic effect on neurons and are the predominant cause of AD development. Impairment in learning and memory processes has been observed to increase with increasing τ-oligomer levels in AD. Synapse loss and microglial activation preceded the formation of neurofibrillary tangles, reflecting disruptions in axonal transport which occur as a result of τ-protein hyperphosphorylation [32].

It is argued that in the case of familial Alzheimer’s disease (FAD), τ-associated pathology should not be considered a downstream link in the amyloid cascade, and that the development of AD, according to the amyloid cascade hypothesis and in the case of tau-associated pathology, follows two independent pathways. Tau-protein hyperphosphorylation first occurs in the brainstem (in the locus coeruleus region), then spreads to the medial temporal lobe, limbic structures, and association and primary cortex. The amyloid formation begins in the association cortex and then develops in the lower regions of the cortex, the brainstem, and the cerebellum [33].

### 2.3. Ubiquitin

Ubiquitin is a small, highly conserved peptide contained in all eukaryotic cells, which is conjugated to proteins that are to be targeted at the proteasome. This process occurs in three steps. First, an ubiquitin monomer is activated in an ATP-dependent reaction by the ubiquitin-activating enzyme (E1). Next, ubiquitin is transported to the ubiquitin-conjugating enzyme (E2). And finally, ubiquitin is transported to the target protein by ubiquitin ligase (E3).

The E3 ligase binds the target protein and the E2-ubiquitin complex, facilitating the formation of a covalent bond between the ubiquitin monomer from the E2 enzyme and the target protein. Activated ubiquitin molecules are successively attached to the first ubiquitin proteins, forming a polyubiquitin chain. Proteins tagged with chains of four or more ubiquitins are recognized by the 26S proteasome for degradation. It is the E3 ligase that imparts specificity to the process, selectively binding to the target protein. Ubiquitin monomers are released after proteasomal degradation or actively removed by ubiquitin carboxyl-terminal hydrolases.

Growing evidence confirms that changes in the UPS function may be involved in the AD pathogenesis process [34]. This standpoint is supported by data confirming that ubiquitin accumulates in both plaques and tangles in AD. Moreover, these structures have been shown to contain a mutant ubiquitin-B protein (UBB+1), mutant ubiquitin carrying a 19-amino acid C-terminal extension formed by a transcriptional dinucleotide deletion.

Remarkably, UBB+1 has been found to inhibit ubiquitin-dependent proteolysis in neuronal cells, inducing the formation of mitochondrial neuritic beads in association with neuronal differentiation, and it is suggested to be a mediator of Aβ-induced neurotoxicity [34].

### 2.4. Alpha-Synuclein

Alpha-synuclein is a small neuronal protein located mostly in presynaptic terminals. It is detected in various parts of the brain, primarily in the neocortex, hippocampus, and substantia nigra. It is also present in other brain cells—astrocytes and oligodendrogliocytes.

Alpha-synuclein accounts for approximately 1% of the total soluble protein pool in the brain. Alpha-synuclein is also found in other cell types, such as blood cells.

The gene encoding the alpha-synuclein protein (SNCA) is located on chromosome 4 (locus 4q21) and consists of six exons, five of which are transcribed. Alternative splicing produces three protein isoforms (140 amino acids, 126 amino acids, and 112 amino acids), of which the 140 isoform is the major one [35].

A protein called alpha-synuclein was initially detected in stingray electroreceptors in screening for synaptic proteins. Human alpha-synuclein was first isolated from amyloid deposits in the frontal cortex of individuals with typical clinical and neuropathological manifestations of Alzheimer’s disease. Alpha-synuclein was later found to be a major component of Lewy bodies in Parkinson’s disease (PD).

The major isoform of alpha-synuclein (140 amino acids, 19 kDa) consists of an amino-terminal region containing several repeats of amino acid sequences (KTKEGV), the hydrophobic central region known as the non-amyloid component (NAC), and the negatively charged acidic C-terminal region. The C-terminal region contains several phosphorylation sites (Tyr-125, -133, -136, and Ser-129), as well as a domain responsible for alpha-synuclein chaperon activity (bases 125–140).

The N-terminal region is very similar to the lipid-binding domain of apolipoproteins, which suggests that alpha-synuclein can interact with the lipid membrane raft. It has been shown to interact with the vesicular membrane containing phospholipids. It is believed that the nucleus accumbens (NAc) is responsible for its fibrillization, while the C-terminal region (bases 96–140) has an inhibitory effect on fibril formation.

Alpha-synuclein is thought to exist in two equilibrium states in the cell: native and membrane-bound. In its native form, it is a soluble, unfolded protein with a weakly ordered or completely disordered structure, lacking a definite spatial organization.

Alpha-synuclein binding to membranes is followed by conformational transition to an alpha helix. Alpha-synuclein is a protein capable of aggregation. An increased concentration of alpha-synuclein in solution leads to the formation of fibrils and discrete spherical structures similar to those present in Lewy bodies.

Some hypothetical functions of alpha-synuclein have been described so far, but the exact physiological significance of this protein remains unknown. The protein’s localization in presynaptic terminals and its ability to interact with membranes suggest that it is involved in the regulation of neuronal vesicular transport.

Vesicle pool depletion in hippocampal synapses observed in SNCA knockout mice allowed to suggests alpha-synuclein involvement in maintaining the presynaptic vesicle pool.

It has also been shown that alpha-synuclein can affect intracellular dopamine amount through direct interaction with proteins that regulate its synthesis and reuptake. By regulating the amount of dopamine transporter in the plasma membrane, alpha-synuclein may act as a regulator of dopamine toxicity by controlling dopamine entry into and exit from the cell.

Alpha-synuclein has been found to affect dopamine synthesis by inhibiting the rate-limiting enzyme in the synthesis of dopamine, tyrosine hydroxylase. Interestingly, SNCA knockout mice do not exhibit significant CNS dysfunction and show no signs of neurodegeneration. Only a triple knockout of alpha-, beta-, and gamma-synuclein genes is accompanied by changes in the synaptic structure, impaired neurotransmission, and age-related neurodegeneration. This observation suggests that the neurodegeneration mechanism in PD may not be related to the loss of alpha-synuclein activity. Overexpression of alpha-synuclein in transgenic mice results in reduced dopamine release and synaptic dysfunction.

As noted above, the amplification of the normal SNCA gene sequence, resulting in increased intracellular alpha-synuclein levels, is sufficient for the development of PD. The disease onset age and severity correlate with the number of gene copies. Alpha-synuclein filaments appear to be the main ultrastructural component not only of Lewy bodies in PD associated with SNCA amplification, but also in sporadic PD and other synucleopathies.

In particular, these protein aggregates were identified in Lewy bodies, which are found in neurons of patients with Lewy body dementia and in glial cytoplasmic inclusions that form in oligodendrocytes in patients with multiple system atrophy.

Allelic variants in the promoter region of the SNCA gene, associated with increased gene expression, increase the risk of developing PD. Furthermore, three independent genome-wide scanning studies have found an association of the SNCA gene locus with an increased PD risk. Alpha-synuclein neurotoxicity has been convincingly confirmed by the creation of transgenic animals (Drosophila, mice) based on overexpression of the human SNCA gene, which exhibit neuronal alpha-synuclein-positive inclusions and the degeneration of dopaminergic neurons in the brain. The neurotoxicity of alpha-synuclein aggregates has also been repeatedly demonstrated in vitro [18].

The ability of alpha-synuclein to form fibrils in vitro, similar to those observed in Lewy bodies, and the fact that the A53T mutation accelerates fibril formation, suggest that alpha-synuclein polymerization may be directly associated with the pathogenesis of PD.

Despite a lot of data pointing to the pathogenic role of fibrillar alpha-synuclein in cells, the mechanisms of fibril toxicity remain unknown.

For the time being, the prevailing hypothesis is that it is not alpha-synuclein fibrils themselves that are toxic, but rather certain intermediates (called protofibrils) which appear in the process of their formation. Protofibrils are small oligomers that have a p-pleated structure. Two types of protofibrils have been observed in vitro: spherical or ring-shaped protofibrils and tubular protofibrils.

Interestingly, post-translational modifications of alpha-synuclein (oxidation, phosphorylation, nitrosylation) enhance its ability to form aggregates. It has been shown that alpha-synuclein found in Lewy bodies is predominantly phosphorylated at Ser129. The mechanisms of neurotoxicity of alpha-synuclein protofibrils are unclear. It has been suggested that protofibrils can form pores capable of building into the membrane, altering its permeability and, consequently, cellular homeostasis. Recent findings point to prion-like properties of alpha-synuclein.

The ability of alpha-synuclein and its aggregates to be secreted and subsequently taken up by neighboring cells has been demonstrated. It is hypothesized that exogenous fibrillar alpha-synuclein may further act as a site for aggregation of soluble monomeric protein. The above discussed data support the hypothesis of the neurotoxicity of fibrillar forms of alpha-synuclein and their role in the pathogenesis of PD, but a precise mechanism of neurodegeneration remains unknown.

### 2.5. DRP1

The involvement of DRP1 in mitochondria and its role in maintaining their shape, size, and distribution appear to be crucial for normal cellular functioning. Electron and confocal microscopy, gene expression analysis, and biochemical methods have helped to demonstrate that neurons from samples treated with Aβ alone exhibit increased expression of DRP1 and Fis1 (fission genes) and decreased expression of Mfn1, Mfn2, and Opa1 (fusion genes), which suggests abnormal mitochondrial dynamics in brain neurons in AD patients [31].

Several research groups have reported that impaired mitochondrial dynamics is associated with abnormal DRP1 expression in postmortem brains of AD patients, AD mouse models, and APP cell lines. Mitochondrial abnormalities in AD have been studied by Hiraiet et al. [36] using in situ hybridization with mtDNA, immunocytochemistry, and electron micrograph morphometry of biopsy specimens from AD patients. They found that the same neurons exhibiting increased oxidative damage in AD had a striking and significant increase in mtDNA and cytochrome oxidase.

Remarkably, the mtDNA and cytochrome oxidase were predominantly found in the neuronal cytoplasm and in lipofuscin-associated vacuoles, which points to the existence of mitochondrial abnormalities and autophagosomes in neurons from AD patients.

There is evidence of abnormal mitochondrial dynamics in primary neurons from AβPP transgenic mice, in brain tissue of transgenic mice (Tg2576 mice) and in brain tissue from AD patients (autopsy), as well as in neuroblastoma cells treated with Aβ peptide.

### 2.6. Markers of Vascular Dementia: Endothelin-1 and CD34 Protein

Endothelin is a biologically active bicyclic polypeptide with a broad spectrum of activity. Today, endothelin is considered one of the most significant regulators of the functional state of vascular endothelium. Information about endothelin as a vasomotor activity factor first appeared in 1985, when a group of researchers led by Hickey studied cultured bovine aortic endothelial cells. They succeeded in purifying this factor and determining the amino acid sequence of the peptide, which consisted of 21 amino acid residues with four cysteine bridges in the form of disulfide bonds and with a molecular weight of 2492 Da.

The extracted factor was named endothelin, and in 1988, an article was published on a peptide that is produced by endothelial cells and has a powerful vasoconstrictor effect. This important discovery was followed by numerous experimental studies using endothelin [37].

However, when the first research results began to appear, it became clear that the mechanism of this peptide action was not as simple as it was initially thought.

A year later, intensive research led to the discovery of three endothelin isoforms: endothelin-1, endothelin-2, and endothelin-3. It was found that all these isoforms consist of 21 amino acid residues, and their synthesis is encoded by three different genes present only in vertebrates, which enables scientists to trace the evolution of endothelins. Moreover, endothelin-2 has a homology very similar to that of endothelin-1 and structurally differs by only two amino acid residues [37].

The polypeptide is known to be produced by endothelial cells as a precursor (preproendothelin), which is then converted into big-endothelin through cleavage of oligopeptide fragments. Big-endothelin consists of 39 amino acid residues. Endothelin-1 is formed by an endothelin-converting enzyme, which is localized inside and on the surface of the endothelium. In this process, the vasomotor activity of endothelin-1 increases by 140 times, and its half-life is shortened. The half-life of endothelin-1 lasts from 40 s to 4–7 min, and a larger part of endothelin (80%) is inactivated while passing through the pulmonary vessels.

Endothelin-1 is the most common member of the endothelin family and the most potent vasoconstrictor, 10 times more powerful than angiotensin II, and its effect is 100 times higher than that of noradrenaline [38].

Amino acid sequencing revealed that this protein has a strong similarity to a toxic component of the venom of spiders and some snakes. In particular, a peptide (sarafotoxin) derived from the venom of the *Atractaspis engaddensis* snake has structural and functional similarities to endothelins. When sarafotoxin is released into the victim’s bloodstream, it causes coronary spasm, which may result in cardiac arrest.

However, there are minor differences in the chemical structure of the venom and endothelin, which are significant for understanding the binding of ligands to endothelin receptors.

Endothelin-1 is predominantly produced in endothelial cells and, unlike other endothelins, can be synthesized in underlying vascular smooth muscle cells, neurons, astrocytes, endometrium, hepatocytes, mesangiocytes, Sertoli cells, mammary endothelial cells, and tissue basophils.

Endothelin-2 is found in the kidneys, intestine, myocardium, placenta, and uterus. Endothelin-3 is localized in the brain, intestine, kidneys, and lungs. Endothelin-1 does not accumulate in endothelial cells, but is rapidly formed under the impact of adrenaline, angiotensin II, vasopressin, thrombin, cytokines, and mechanical action.

In a few minutes, mRNA transcription is activated and endothelin precursors are synthesized, followed by their conversion to endothelin-1 and subsequent secretion. At the same time, catecholamines, angiotensin II, high-density lipoproteins, growth factors, thrombin, thromboxane A2, Ca^2+^ ionophore, and phorbol ester activate intracellular mechanisms of endothelin-1 synthesis, bypassing cell membrane receptors by influencing protein kinase C and releasing Ca^2+^ from the sarcoplasmic reticulum. Hypoxia in some tumors also leads to endothelin production, which, in turn, results in disease progression.

The concentration of endothelin-1 in human blood plasma is normally 0.1–1 fmol/mL or undetectable. It is the concentration level that determines what effect (relaxation or contraction) will be produced. At low concentrations, endothelin acts on endothelial cells in an autocrine/paracrine manner, releasing relaxation factors, while an increased concentration activates receptors on smooth muscle cells in a paracrine manner, leading to vascular spasm. One of the most significant regulators of endothelin production in endothelial cells is the transforming growth factor (TGF-β), which increases the production of preproendothelin [38].

As far as we now know, the vasoconstrictor effect, increased heart rate and contractility (chronotropic and inotropic effects) of endothelin-1, as well as potentiating tissue growth and differentiation, are realized through the activation of two types of receptors: ETA and ETB. ETA has a high affinity for endothelin-1 and endothelin-2. ETB does not act preferentially, but it does have two subtypes: ETB1 and ETB2. About ten years ago, another type of endothelin receptor, ETC, was identified. Its structure and role are not yet fully understood. However, endothelin-3 is believed to act through ETC receptors.

Receptor subtypes are differently localized in the vascular system: ETA is found in vascular smooth muscle cells, cardiomyocytes, brain tissue, and the gastrointestinal tract. ETB is found in smooth muscle cells, coronary vessels, cardiomyocytes, juxtaglomerular cells, and the ileum.

Gender also plays a significant role. It has been proven that ETA activation is increased in males, while ETB activation has been observed in females. The vascular lumen diameter is significant, too. ETA is expressed in small-diameter vessels, while ETB is predominantly expressed in coronary arteries and pulmonary vessels. The different localization of the receptors allows us to explain a large number of effects associated with endothelin function under normal and pathological conditions.

### 2.7. PTEN-Induced Kinase 1 (PINK1) and Parkin

PTEN-induced kinase 1 (PINK1) is involved in determining the degree of mitochondrial damage. This protein contains an amino acid sequence that attracts it to mitochondria. Normally, PINK1 kinase is involved in the phosphorylation of mitochondrial proteins and alters their functional activity. Homozygous mutations in the PINK1 gene cause a loss of PINK1 kinase enzymatic activity, production of defective ATP, and excessive formation of free radicals. The significance of heterozygous mutations in this gene is not fully understood; however, according to the findings of numerous studies, heterozygosity may be a risk factor for late-onset PD. 

In normal mitochondria, PINK1 is imported through the outer mitochondrial membrane via the TOM complex and partially passes through the inner mitochondrial membrane via the TIM complex, stopping in a position that spans the inner mitochondrial membrane.

Import through the inner membrane is accompanied by PINK1 cleavage, reducing its weight from 64 kDa to 60 kDa. Subsequently, with the involvement of the PARL protein, it is converted into a 52 kDa protein. The new form of PINK1 is degraded by mitochondrial proteases. Thus, PINK1 concentration is under control in healthy mitochondria [20].

In damaged mitochondria, the inner mitochondrial membrane becomes depolarized. This membrane potential is important for TIM-mediated protein import. In the case of a depolarized membrane, PINK1 cannot pass through the inner mitochondrial membrane and is not cleaved by the PARL protein, and therefore, the concentration of PINK1 in the outer membrane increases. PINK1 may then recruit Parkin. PINK1 is thought to phosphorylate ubiquitin attached to the serine 65 residue of Parkin, resulting in Parkin being recruited to mitochondria.

Parkin is a cytosolic E3 ubiquitin ligase. Once Parkin enters mitochondria, PINK1 phosphorylates Parkin at residue S65 (the same residue where ubiquitin was attached). The result of this phosphorylation is that Parkin is dimerized and activated, acquiring the ability to bind ubiquitin to other proteins. Since PINK1 recruits Parkin to the mitochondrial surface, Parkin can ubiquitinate proteins on the outer mitochondrial membrane, including such proteins as Mfn1/Mfn2 and mitoNEET. Ubiquitinylation of the mitochondrial surface is one of the factors that trigger mitophagy.

Parkin triggers the addition of ubiquitin chains to lysine residues K63 and K48. Ubiquitinylation at K48 triggers protein degradation and can lead to passive destruction of mitochondria. Ubiquitinylation at K63 leads to the recruitment of the mitophagy adaptor proteins MAP1LC3A/GABARAP, which eventually triggers mitophagy. It is unknown which proteins are necessary and sufficient for mitophagy and how these proteins, when ubiquitinated, trigger mitophagy [18].

Mutations in the PINK1 gene are the second most common after Parkin-associated forms of PD, being clinically very similar to this type of disease. The PINK1 gene is localized on the short arm of chromosome 1 at locus 1p35–36 and encodes mitochondrial serine/threonine protein kinase 1 (PTEN-induced kinase 1). The PINK1-associated form of PD accounts for approximately 4–7% of sporadic cases of the disease. The most common in the PINK1 gene is p.Gln456Ter point mutation. However, approximately 111 other missense and nonsense mutations, insertions, splice site mutations, and large deletions have been described [39].

### 2.8. NF-kB Transcription Factor

The transcription factor NF-κB (nuclear factor kappa B, also known as nuclear factor kappa-light-chain-enhancer of activated B cells) is a ubiquitous transcription factor that controls the expression of genes involved in immune response, apoptosis, and cell cycle. NF-kB dysregulation may cause inflammation, autoimmune diseases, and the development of viral infections and cancer. The NF-kB family consists of five proteins: NF-kB1 (or p50), NF-kB2 (or p52), RelA (or p65), RelB, and c-Rel, which form 15 combinations of dimers. All proteins in the NF-kB family share a Rel homology domain, which mediates the protein dimerization and binding of NF-kB to DNA and to the cytosolic inhibitory protein IkB. NF-kB is active only in its dimeric form (formation of both hetero- and homodimers is possible), and the most common forms are dimers of the p50 or p52 subunit with the p65 subunit [40].

NF-kB can be activated by various stimuli, including cytokines (such as TNF and interleukin 1), T- and B-cell mitogens, bacterial and viral products (all toll-like receptor ligands, such as lipopolysaccharide or double-stranded viral RNA), and stress factors (such as reactive oxygen species or ultraviolet radiation). NF-κB is found in the cell’s cytoplasm in an inactive form as a complex with the inhibitory protein IκB.

A stimulating agent activates the NF-κB signaling pathway, and IκB is catalyzed under the action of IKK (IκB kinase), which results in IκB degradation by the 26S proteasome. At the same time, NF-κB is released from the inhibitory complex, translocates to the nucleus, and activates transcription of the genes under its control.

Neuroinflammation is a common feature of several CNS diseases, characterized by upregulation of proinflammatory cytokines and chemokines such as *Tnf*, *Ccl2*, and *Cxcl10*, as well as infiltration of activated immune cells.

Activation of the NF-κB family of transcription factors is a key stage in the regulation of inflammatory and immune responses. However, these proteins also regulate gene expression in many other physiological processes, such as cell proliferation, differentiation, and survival, as well as specific functions of the central nervous system, including learning and memory. In resting cells, NF-κB dimers are sequestered in the cytosol by inhibitory proteins of the IκB family. A crucial step in NF-κB activation is the phosphorylation of IκB proteins by the IκB kinase activating complex. IKK2 is a critical subunit of the kinase that induces a canonical signaling pathway, which is essentially involved in inflammation regulation. 

The phosphorylation of IκB inhibitory proteins initiates their ubiquitinylation and subsequent proteasomal degradation, followed by the release and nuclear translocation of active NF-κB dimers, which then induce the expression of NF-κB target genes 49 [40].

The IKK/NF-κB signaling system is supposed to be involved in the pathogenesis of various neurological diseases. It has been characterized as a central regulator of inflammatory responses, controlling the expression of numerous genes affecting inflammation. 

The IKK/NF-κB system is thought to play a dual role in the pathogenesis of neurological disorders. Due to its proinflammatory function, NF-κB activation can cause neuronal dysfunction, aging, and cell death, thereby increasing the severity of CNS diseases. 

Conversely, NF-κB activation can also mediate neuroprotection, as IKK/NF-κB signaling plays a crucial role in neuronal differentiation and various CNS functions. However, due to its complex regulation in different cell types and diverse responses to various physiological and pathological conditions, the function of the IKK/NF-κB system in CNS physiology and pathology remains unclear in detail [10].

### 2.9. RAGE Protein

The RAGE protein plays a key role in the pathogenesis of neurodegenerative diseases. This protein is a receptor for advanced glycation end products (AGEs), a type 1 transmembrane glycoprotein of the immunoglobulin family. It regulates such functions as neuronal growth, survival, and regeneration, and also mediates the innate immune response and induces the production of cytokines and chemokines.

The RAGE receptor is expressed in all endothelial cells, smooth muscle cells, T- and B-lymphocytes, but higher levels of expression are observed in neurons of the central nervous system. When its expression is impaired, it is involved in the activation of neurodegenerative diseases, especially AD.

Dysregulation of RAGE protein expression is linked with such factors as increased ligand concentrations (beta-amyloid, S100, AGEs), effects on the receptor of the hypoxia-inducible factor, and increased levels of insulin in blood. There are several types of RAGE proteins: esRAGE, sRAGE, and mRAGE.

The esRAGE (endogenous secretory RAGE) is involved in the pathogenesis of AD through the interaction of m-RAGE with beta-amyloid or through inhibition of the mRAGE signaling pathway. Plasma esRAGE levels can mark the severity of AD. As AD develops, RAGE expression is detected in cells, including senile plaques, such as glial cells, neurons, and endothelial cells.

The primary ligands of the RAGE receptor involved in neurodegenerative processes are AGEs, Aβ, and S100. RAGE activation by AGEs or Aβ can increase the expression of BACE 1, a key enzyme promoting Aβ production in the brain. AGEs are formed through the non-enzymatic reaction of proteins and lipids with sugars. The interaction of AGEs with RAGE stimulates the formation of reactive oxygen species by activating NADPH oxidase, and the resulting ROS is involved in the early toxic events that lead to AD progression [41].

Beta-amyloid is a peptide consisting of 142 amino acids, which is formed as a result of proteolysis of the APP protein. Aβ plays a key role in the formation of intracellular inclusions, i.e., neuronal plaques, which lead to the development of degenerative processes in neurons and subsequently to cell necrosis or apoptosis.

RAGE is involved in the Aβ-dependent attenuated synaptic transmission, contributing to the inhibition of synaptic plasticity. Aβ and AGEs reduce neuronal mitochondrial activity and induce neurodegeneration through mitochondrial dysfunction. sRAGE interacts with Aβ in the blood–brain barrier membrane and facilitates this protein transport.

Increased Aβ concentrations bind to RAGE, specifically the 60–76 region of RAGE, stimulating activation of the NF-κB transcription factor, which is involved in the cellular response to neurodegeneration. Prolonged RAGE activation can lead to cellular dysfunction.

Another RAGE receptor ligand is the S100 protein, which is also involved in neurodegenerative processes by activating glia, which results in neuronal dysfunction. S100 induces degenerative changes in axons and promotes the growth of degenerative axons expressing APP. It is through proteolysis of APP that the beta-amyloid involved in AD development is formed [42].

### 2.10. S100 Protein

The S100 protein was first described in 1965 as a fraction of neuroglial proteins produced primarily by astrocytes in the brain. The cerebral S100 protein is a combination of two closely related proteins of the family: S100A1 (S100α) and S100B (S100β).

By 2004, 20 members of the S100 family (intracellular calcium-sensor and calcium-binding proteins with molecular weights of 10–12 kilodaltons) had been discovered.

Of the 20 genes that encode the synthesis of S100 proteins in humans, 16 are located in the q21 region of chromosome 1. These genes are designated S100A (1, 2, …, 16). The S100B gene is located in the q22 region of chromosome 21.

S100 family proteins exist as dimers inside the cell. So, S100A1 and S100B in the brain form homodimers S100A12 and S100B2, as well as heterodimers S100A1/S100B [33].

Due to their ability to regulate the activity of a wide range of proteins, S100A1 and S100B are involved in the transduction of signals that control the activity of energy metabolism enzymes in brain cells, calcium homeostasis, the cell cycle, cytoskeletal functions, transcription, cell proliferation and differentiation, cell motility, secretory processes, and the structural organization of biomembranes.

However, the most unusual characteristic of some members of the S100 family is their ability to be secreted extracellularly. S100 proteins in the extracellular sector manifest cytokine properties and interact with RAGE receptors, which are expressed in the nervous system by neurons, microglia, astrocytes, and vascular wall cells [20].

Research findings over the past decade have demonstrated that glial cells not only provide structural support and nutrition for neurons but also interact intensively with them. Due to the presence of ion channels, as well as receptors for neurotransmitters and other signaling molecules in their distal parts, astrocytes are able to detect changes in neuronal activity and respond to them by increasing cytosolic calcium concentrations and generating calcium waves.

The calcium signal is then realized (possibly with the direct involvement of the S100 protein) into gene expression modulation, changes in astrocyte morphology, and the secretion of some neuroactive molecules, such as glutamate, D-serine, ATP, taurine, neurotrophins, and cytokines.

Astrocytes perform a wide range of adaptive functions, including neurotransmitter reuptake, contributing to damage repair, and regulating synaptic density. These findings provide evidence that glia–neuronal reciprocal signaling, functional and structural plasticity play a major role in the functioning of neuronal networks and information transmission/processing processes in the nervous system during its formation, functioning, and reparation. One of the mediators in neuron–glial and glia–glial interactions is S100B, which is secreted by glial cells [42].

As in many biologically active molecules, the effects of extracellular S100B are dose-dependent. At nanomolar concentrations, S100B exerts an autocrine effect on astrocytes, stimulating their proliferation in vitro, while the S100B2 dimer modulates long-term synaptic plasticity and exerts a trophic effect on both developing and regenerating neurons.

At micromolar concentrations, extracellular S100B (in its homo- and heterodimeric forms) can exert neurotoxic effects on neurons and glia, inducing both cell apoptosis and cell necrosis. The latter effect is based on the ability of S100B to independently induce proinflammatory cytokines and oxidative stress enzymes, particularly iNOS, and to enhance other signals directed at neurons and glial cells.

Thus, the S100B protein can enhance the expression of interleukin-1 (IL-1) and interleukin-6 (IL-6) in microglia and neurons, which may result in pathological changes in neuronal properties, particularly hyperphosphorylation of tau protein, decreased levels of certain synaptic proteins, and increased synthesis and activity of acetylcholinesterase. S100B also increases the expression of the β-amyloid precursor peptide (APP) and its mRNA in neuronal cultures and enhances β-amyloid peptide-induced activation of astrocytes. In turn, both IL-1 and β-amyloid induce S100B expression, thus perpetuating a vicious cycle of potentiating the neurotoxic effects of S100B.

S100B-induced enhanced APP expression and iNOS activation may contribute to the spreading of inflammatory activation and neurodegeneration, as the β-amyloid peptide can be secreted and nitric oxide (NO) can diffuse. NO, in turn, can trigger the synthesis and release of other neurotoxic molecules from astrocytes, such as IL-8 and tumor necrosis factor alpha (TNF-α).

Many studies have tried to find a link between chronic glial activation (astrocytes and microglia) and subsequent progressive cycles of neuroinflammation, autoimmune reactions, neuronal dysfunction, and neurodegeneration in AD.

There are numerous stimuli that are responsible for chronic inflammatory glial activation, including cytokines (IL-1, TNF-α), lipopolysaccharide (LPS), and β-amyloid-42. The resulting neurotoxic glial products can enhance glial activation and thereby contribute to the progression of chronic neurodegenerative diseases.

One of such potentially neurotoxic compounds is the glial cell product—S100B. S100B synthesis in AD can increase severalfold, with protein levels reaching micromolar concentrations, in comparison with healthy age-matched controls. Moreover, S100B levels increase precisely in those brain regions that are associated with AD pathogenesis.

In the case of AD, S100B levels in the brain are higher due to activated astrocytes, cellular components of amyloid plaques, which contain an increased amount of S100B [42]. S100B is known to stimulate axonal growth and neuroprotection, and its increased levels in the brains of AD patients may initially be a part of a compensatory response. Overexpression of this protein, however, may also exert adverse effects.

The neurotrophic activity of S100B also contributes to aberrant axonal hypertrophy and the formation of large dystrophic neurites in and around amyloid plaques, and chronically elevated S100B levels in the brain result in enhanced expression of APP, which may cause further accumulation of amyloid peptide.

S100B can also stimulate glial activation, leading to neuroinflammation and neuronal dysfunction. The degree of astrocytosis is known to vary among AD patients. Diffuse amyloid plaques are associated with mild astrocytosis, while axonal plaques are associated with a large amount of activated astrocytes.

S100B concentrations may reflect the ratio of these two plaque types in AD, as the number of S100B-overexpressing astrocytes and elevated S100B levels in tissue correlate with the density of neuritic plaques and the density of APP-overexpressing dystrophic neurites inside individual plaques. Thus, S100B overexpression occurs in association with neurodegeneration and may induce a damaging effect [43].

These findings suggest that S100B directly induces degenerative changes in axons and promotes the growth of degenerative axons overexpressing APP in diffuse amyloid deposits, as well as transforming benign diffuse deposits into diagnostic axonal plaques responsible for cortical atrophy in AD.

Increased S100B content in the brain of AD patients is also directly associated with tau-positive neuritic pathology. S100B overexpression, with its subsequent trophic and toxic effects on neurons, may be an important pathogenetic mechanism in the development of neuritic and neurofibrillary pathological changes in AD.

In patients with AD and vascular dementia, parallel overexpression of S100B and the proinflammatory cytokine IL-1 has been observed, which plays a significant role in the pathogenesis of neuropathological changes. An association has been noted between glial cells overexpressing IL-1 and S100B and increased neurofibrillary tangles of tau protein [44].

### 2.11. Claudin

Tight junction proteins of the blood–brain barrier are vitally important for maintaining the integrity of endothelial cells lining blood vessels of the brain. These protein complexes are located in the space between endothelial cells, where they create a dynamic, restrictive, and highly regulated microenvironment that is vital for neuronal homeostasis [11]. 

By restricting paracellular diffusion of material between the blood and brain, tight junction proteins form a kind of protective barrier preventing the passage of unwanted and potentially harmful material [11]. 

At the same time, this protective barrier hinders the therapeutic efficiency of drugs acting on the central nervous system, as more than 95% of small molecule therapeutics are unable to cross the blood–brain barrier [20]. 

Claudin-5 is the most enriched tight junction protein in the blood–brain barrier and its dysfunction is associated with neurodegenerative disorders such as AB, multiple sclerosis and some psychiatric disorders.

Tight junctions are a vital component of the brain endothelium, regulating blood-brain exchange and protecting delicate neural tissue from blood-borne insults such as pathogens and immune cells [21].

Various upstream signaling components can regulate claudin-5 levels, and targeting these pathways to modulate claudin-5 expression in response to certain pathologies is a promising therapeutic strategy. 

Furthermore, RNAi-mediated suppression of claudin-5 is a well-studied strategy for transiently modulating BBB permeability, enabling small-molecule therapeutics in preclinical disease models, which may be useful for the treatment of various CNS diseases [31].

## 3. Other Signaling Molecules

### 3.1. Vascular Peptides

Various studies have identified a number of classical and putative neuropeptides associated with the development and progression of neurodegenerative diseases. Arginine vasopressin, synthesized in the hypothalamus and regulating fluid balance and stress response, has shown dysregulation in the Wernicke-Korsakoff syndrome, a subtype of VD, which correlates with cognitive decline and hippocampal damage. Elevated arginine vasopressin levels in the right parahippocampal gyrus have also been observed in AD patients, but using it as a specific biomarker is restricted by its nonspecificity and the impact of comorbidities [10].

### 3.2. Neuroendocrine Regulators

Gastrin-releasing peptide, which functions through bombesin receptors, plays a dual role: on the one hand, it promotes arterial damage and intimal hyperplasia by enhancing vascular smooth muscle cell proliferation and migration, and on the other hand, it mitigates cognitive impairment in VD patients by modulating neuronal activity in the hippocampus and influencing neurogenesis. Glucagon-like peptides (GLP-1 and GLP-2), proglucagon derivatives, exhibit neuroprotective properties.

### 3.3. Neuroinflammatory Peptides

Adrenomedullin (AM), a multifunctional neuropeptide with vasodilatory, antiapoptotic, and anti-inflammatory effects, is strongly expressed in endothelial cells. Its expression increases after vascular insults, potentially promoting the restoration of vascular function through the activation of growth factors. AM deficiency is associated with VD exacerbation, and its conjugates are considered potential therapeutic agents.

Serum somatostatin and neuron-specific enolase (NSE) have also been proposed as biochemical markers of early VD: decreased somatostatin levels and increased NSE correlate with cognitive deficits. Among the putative neuropeptides, leptin and adiponectin, adipokines produced by adipose tissue, demonstrate a significant association with cognitive functions. Low leptin levels are associated with an increased risk of cognitive impairment and dementia, while adiponectin impacts cerebral vascular function and improves cognitive function in patients with visceral hypertension [45]. Despite significant progress, issues of standardization of peripheral biomarkers and their sensitivity in the early stages remain unresolved. There is also a lack of data on the dynamics of biomarker expression during dementia treatment [45].

A stepwise diagnostic algorithm is used in the clinic: clinical neuropsychological assessment and MRI; if in doubt, biomarker screening is performed in patients with mild cognitive decline or dementia; and if the results are positive or inconsistent, a cerebrospinal fluid (CSF) analysis or PET scan is performed. This approach is consistent with the updated concept of biological diagnosis of AD, where the diagnosis is not only based on symptoms (memory, thinking, behavior), but also on objective biomarkers reflecting pathology in the brain—even when clinical manifestations are minimal or absent [15].

Clinical screening includes the patient’s complaints, objective assessment of cognitive deficits (MMSE, MoCA, CDR-SB scales), assessment of daily functioning, and exclusion of reversible causes of dementia. Neuropsychological assessment remains the first line and determines the feasibility of further biomarker-based diagnosis specification and differential diagnosis [1]. In the case of mild cognitive impairment, neuroimaging and/or fluid-based biomarkers may increase diagnostic confidence and stratify the risk of disease progression [9]. AD diagnostic criteria have been revised to include biomarkers divided into two categories: markers of amyloid pathology (decreased Aβ42 levels in the cerebrospinal fluid, positive PET imaging with amyloid ligands) and markers of neuronal damage/tau pathology (increased tau and phospho-tau in the cerebrospinal fluid, decreased glucose metabolism on FDG-PET, medial temporal lobe atrophy on MRI). The combination of these biomarkers helps to diagnose AD in the preclinical and prodromal stages, long before the manifestation of overt dementia. Genetic risk factors for AD, such as mutations in the APP, PSEN1, and PSEN2 genes (which enhance Aβ42 production) and the ApoEε4 allele (which impairs Aβ clearance), are directly linked to these processes. Biomarkers are recommended as a preliminary screening for amyloid status, followed by cerebrospinal fluid analysis or PET, if necessary [26].

MRI is a mandatory step to exclude alternative pathologies and to assess neurodegeneration (medial temporal lobe/hippocampal atrophy, parieto-temporal changes). Metabolic patterns on PET imaging (bilateral parieto-temporal hypometabolism, posterior cingulate cortex) confirm the diagnosis of AD and help differentiate it. This method is recommended for the early diagnosis of MCI with suspected neurodegeneration [1]. Amyloid and tau PET provide direct confirmation of amyloid and/or tau pathology and are used in clinically complex cases or to confirm the diagnosis when other methods give conflicting results.

Table 1 presents summarized data on the functional role of major biomarkers in neurodegenerative processes of the central nervous system.

## 4. Analytical Platforms for Determining Neurodegenerative Biomarkers

The clinical utility of neurodegenerative biomarkers is determined primarily by the analytical capabilities of the methods used for their quantitative determination in cerebrospinal fluid (CSF) and peripheral blood.

Classical sandwich enzyme-linked immunosorbent assay (ELISA) kits are used to analyze Aβ42, total tau, and phospho-tau in CSF and were the first assays to enter clinical practice. They provide limits of detection (LoD) in the low pg/mL range and remain the standard for diagnosing AD using CSF [46]. Furthermore, the extremely low concentrations of these proteins in plasma have stimulated the development of ultrasensitive platforms.

Immunochemiluminescence assays (ICLA) have better sensitivity and achieve LoDs of approximately 1 pg/mL (detection range from 0.25 to 500 pg/mL), making them suitable for screening studies [25]. Automated ICLA analyzers allow for the parallel determination of Aβ42, total tau, and p-tau181 with LoDs in the low pg/mL range, providing standardized, highly sensitive testing [47].

Digital immunoassay platforms such as Simoa^®^ offer sensitivity up to 1000-fold higher than ELISA, reaching femtogram levels. For example, Simoa can detect Aβ1–42 and Aβ1–40 with LoDs of ~1.5 and ~4.1 pg/mL, respectively, and p-tau181 at LoDs of up to 0.34 pg/mL, enabling the reliable measurement of low plasma biomarker concentrations [48].

Mass spectrometry methods, particularly immunoprecipitation coupled with tandem mass spectrometry (LC–MS/MS), provide high specificity and have been successfully used for the quantitative analysis of plasma Aβ42/Aβ40. Such methods, with a linearity range of approximately 10–2500 pg/mL, can reduce the need for PET [49,50]. New biosensor platforms continue to reduce LoD to femtogram and even attomolar levels. Optical and electrochemical immune sensors for p-tau181 exhibit LoDs of approximately 1.9 fg/mL while maintaining a wide dynamic range [51]. Label-free optical biosensors based on double prisms and silicon structures achieve LoDs of 12 fg/mL and provide a range of 0.1 pg/mL to 10 ng/mL when detecting, for example, Aβ1–42 in blood [51].

Combined methods such as surface-enhanced Raman spectroscopy (SERS) and machine learning enable the simultaneous quantification of Aβ40, Aβ42, total tau, and p-tau in a single plasma droplet [28]. Biomarkers isolated from exosomes and extracellular vesicles are attracting increasing attention: tau, p-tau, synaptic proteins, inflammatory mediators specific to neurons and astrocytes, which allows for obtaining a specific “trace” of intracerebral pathology when analyzing peripheral blood [52]. Review studies show that such markers have the potential to change the current diagnostic paradigm of AD (Table 2).

## 5. Comprehensive Molecular Biomarker Panels

The relevance of research on biomarkers of neurodegenerative diseases is driven by the need for a deeper understanding of the molecular and cellular mechanisms underlying their development [26]. A promising direction is the search for less invasive approaches to studying molecular changes compared to CSF analysis. Particular attention is paid to the integration of data on various classes of signaling molecules—neuropeptides, pathological proteins (Aβ, tau), markers of neuroinflammation, oxidative stress, synaptic dysfunction, and genetic risk factors—for a comprehensive characterization of pathophysiological processes in dementia [4]. This will not only improve the accuracy of differential diagnosis between different types of dementia (e.g., VC and AD, which often coexist), but also ensure patient stratification for personalized therapy and the objective assessment of the effectiveness of new pharmacological agents in clinical trials.

Given that the development of standardized, highly sensitive, and specific biomarker detection methods remains an important task for their widespread implementation in clinical practice, we studied combinations of molecular markers whose expression patterns differ between patient groups and may reflect different pathophysiological mechanisms. An immunocytochemical (ICC) study was conducted to identify signaling molecules (molecular markers) involved in the neurodegeneration process in buccal epithelial cells and peripheral blood lymphocytes in patients with vascular dementia due to AD, as well as in age-matched volunteers without this pathology.

The study was carried out on a cohort of 203 participants, divided into three groups: a group of patients with clinically diagnosed AD (57 persons, 21% male and 79% female); a group of patients with clinically diagnosed VD (100 persons, 26% male and 74% female); and volunteers without clinical manifestations of neuropsychiatric disorders (46 persons, 32.6% male and 67.4% female). The average age of the examined patients in all the groups was 84.6 ± 7.6 (with AD—77 ± 12.2 years, with vascular dementia—79.7 ± 9.8 years, and volunteers—61.7 ± 7.6).

Cases of dementia, including Alzheimer’s disease and vascular dementia, were identified through an analysis of medical records using strict diagnostic algorithms in accordance with current European (EFNS-ENS Guideline on diagnosis and management of disorders associated with dementia) and national recommendations [1]. The diagnosis of vascular dementia was additionally based on the criteria of the Vascular Impairment of Cognition Classification Consensus Study (VICCCS), which provides for a comprehensive assessment of the clinical syndrome, neuroimaging signs of cerebrovascular disease, and their causal relationship with cognitive decline. The methodology for selecting study participants consisted of an expert assessment of medical records (including analysis of diagnoses, anamnestic data, results of biochemical, functional, tomographic studies, and psychometric tests), an assessment of geriatric status based on a comprehensive geriatric assessment, and, if necessary, additional examination and testing according to expert recommendations.

Biological samples included peripheral blood lymphocytes and buccal epithelial cells, selected as relevant extracerebral proxy markers of neurodegeneration. Venous blood was collected using vacuum systems with EDTA anticoagulant under aseptic conditions on an empty stomach. Samples were stored at 2–8 °C for ≤4 h. The lymphocyte fraction was isolated by centrifugation on a Ficoll gradient (1.7 g/mL density, 2000 rpm, 20 min) followed by double washing with Hanks’ solution. Sterile cytobrushes were used for buccal epithelium: after rinsing the mouth, spiral scrapings were taken from the inner surface of the cheeks, fixed in buffer solution, and stored at 2–8 °C for ≤14 days. For the ICC analysis, monolayer cytopreparations were prepared from both types of biomaterials on glasses with L-polylysine coating using a CytoPrep-4 cytocentrifuge (1500 rpm/6 min for lymphocytes, 1000 rpm/6 min for epithelium) with fixation with cold ethanol. The expression of 11 signaling molecules associated with neurodegeneration was studied: Tau protein, β-amyloid, α-synuclein, DRP1 and CD34 proteins, endothelin-1, transcription factor NF-kB, PTEN-induced kinase 1 (PINK), RAGE and S100 proteins, and claudin-5. For visualization of the ICC test, a reagent kit based on NovoLink polymer and peroxidase, RE7150-K, Novo Castra, was used. The ICC protocol included standardized steps: antigen retrieval, blocking of endogenous peroxidase and non-specific binding, incubation with primary antibodies (60 min, room temperature), post-priming with polymeric secondary antibodies (30 min), DAB staining (5 min), and nuclear counterstaining with Mayer’s hematoxylin.

Quantitative assessment of marker expression was performed using computer morphometry. A Leica Aperio AT2 histological scanner was used for lymphocyte analysis, followed by analysis in Aperio ImageScope 12.1 software. Automated segmentation was performed using the OpenCV library (Python3.14.2) and verification in ImageJ 1.52u. Images of buccal epithelial slides at 400× magnification were obtained using two computer-aided microscopic image analysis systems. The first consisted of an Olympus IX73 microscope, an Olympus DP80 digital camera, an AMD Ryzen 3 3200G-based personal computer, and CellSens software (version 4.2CS-ST-V4.2). The second system included an Olympus BX46 microscope, a VideoZavrStandartVZ-18C23-B digital camera, an AMD Ryzen 3 3200G-based personal computer, and VideoZavrCatalog 2.3. software. The number of immunopositive cells and the total cell count were assessed manually. The so-called “specific gravity of cells” (the ratio of the number of immunopositive cells to the number of all cells in the field of view, expressed as a percentage) was also determined with a total number of cells in the micropreparation of at least 100.

Statistical analysis of data for normal distribution was performed using the Shapiro–Wilk test. The results are described using parametric and nonparametric methods. If the data corresponded to the normal distribution law (only for age in the comparison groups), the typical value was presented as the mean and standard deviation (M ± SD), and comparison of groups was performed using Student’s t-test. If the data did not correspond to the normal distribution law (all extensive indicators characterizing the proportion of cells expressing markers), the typical value was presented as the median, the spread was characterized by the interquartile range—Me (Q1-Q3), 95% confidence interval was calculated using the Wilson method. Group comparison was performed using the Mann–Whitney test. The null hypothesis was rejected at a significance level of less than 0.05.

This comprehensive study allowed to identify and confirm the expression of key signaling molecules in peripheral biological tissues (blood lymphocytes and buccal epithelial cells), which are directly associated with the development of dementia of different origins.

Table 3 presents data from the comparative analysis of l1 biomarkers in the buccal epithelium and peripheral blood lymphocytes in patients with AD, vascular dementia, and healthy controls.

Analysis of these data allowed us to identify differences in the expression of a number of molecular markers associated with cognitive impairment and various neurodegenerative processes, taking into account the mechanisms of their development.

Our findings are consistent with the pathogenetic changes in dementia described in the review. For example, decreased expression of RAGE protein, PINK1, and α-synuclein in the buccal epithelium of patients with AD reflects impaired mitochondrial function and proteinopathies. Increased DRP1 in lymphocytes is consistent with the known hyperactivation of mitochondrial function in AD and diabetes. Decreased claudin-5 in the buccal epithelium in diabetes is consistent with impaired endothelial barrier function described in the literature.

The study results indicate that peripheral blood lymphocytes and buccal epithelial cells can be considered as promising biological material for studying molecular changes associated with neurodegenerative processes. The buccal epithelium deserves special attention, as it offers significant advantages thanks to easy and safe noninvasive sampling and a broader spectrum of molecular markers detectable in it as compared to blood lymphocytes.

The main findings of the study are summarized below:

1. The DRP1 protein expression levels in blood lymphocytes were statistically significantly increased, while the expression levels of β-amyloid, NF-κB and tau protein in the buccal epithelium were statistically significantly decreased both in AD patients and in patients with vascular dementia as compared to healthy controls.
2. Expression of α-synuclein, RAGE and PINK1 proteins in the buccal epithelium showed a statistically significant decrease in AD patients as compared with both VD patients and the control group.
3. The claudin expression levels in the buccal epithelium were statistically significantly decreased in patients with vascular dementia as compared to healthy controls.
4. Opposite changes in S100 protein expression were observed: its level in blood lymphocytes was significantly elevated, while in the buccal epithelium, it was significantly decreased in AD patients as compared to the control group.

The analysis revealed statistically significant differences in the expression of several biomarkers, allowing us to identify two sets of marker combinations reflecting different pathogenetic mechanisms. One combination was associated with molecular changes observed in dementia as a clinical syndrome, while the other was associated with molecular patterns that differ between dementia subtypes. These results should be considered hypothetical rather than diagnostic.

Based on the combined results of the study, two complexes of molecular patterns were identified, reflecting different pathogenetic mechanisms:

(a) “Combinations of molecular markers associated with cognitive impairment”, which includes analysis of expression of DRP1 and S100 proteins in blood lymphocytes, and β-amyloid, NF-κB, tau protein, claudin, and S100 protein in the buccal epithelium (Table 4). This combination of markers is associated with molecular changes detected in patients with dementia and may be informative for further prognostic studies.
(b) “A combination of molecular markers associated with Alzheimer’s disease, based on the expression patterns of α-synuclein, RAGE, PINK1, and tau protein in buccal epithelium. This complex reflects molecular differences between dementia subtypes and should not be interpreted as a validated diagnostic tool. (Table 5).

Future research may focus on developing integrative analytical approaches aimed at a more detailed characterization of the variability of molecular changes associated with asthma. The use of additional prognostic methods may facilitate a more accurate interpretation of molecular patterns and their relationship to the biological processes of the disease. However, the clinical applicability of such approaches requires separate prospective validation.

## 6. Conclusions

Neurodegenerative diseases, including Alzheimer’s disease and vascular dementia, represent a serious and growing global health problem. Their complex and multifactorial pathogenesis involves the interaction of mechanisms such as protein aggregation, mitochondrial dysfunction, neuroinflammation, endothelial dysfunction, and impaired blood–brain barrier permeability. In this context, identifying the molecular changes associated with these processes remains an important scientific challenge for advancing the biological understanding of cognitive impairment.

The search for biomarkers in vascular dementia and Alzheimer’s disease is of great importance for modern neurology and cognitive medicine. Biomarkers can identify biological changes that precede pronounced clinical manifestations and contribute to a deeper understanding of the mechanisms underlying the development of neurodegenerative diseases.

This paper summarizes molecular biomarkers whose differential expression is associated with cognitive impairment and various neurodegenerative processes.

Our findings of decreased expression of RAGE, PINK1, α-synuclein, and claudin-5, as well as increased expression of DRP1 and S100 in peripheral cells, are consistent with the pathophysiological mechanisms described in the literature. Based on these results, two complexes of molecular patterns associated with different pathogenetic mechanisms were identified. This study was not designed to evaluate their prognostic accuracy, sensitivity, or specificity, and therefore, no conclusions regarding clinical diagnostic efficacy can be drawn.

Research in this area may help to identify and confirm the expression of key signaling molecules in peripheral biological tissues (blood lymphocytes and buccal epithelial cells), which are directly associated with the development of dementia of different origins. The obtained data convincingly demonstrate that peripheral blood lymphocytes and buccal epithelial cells are highly informative biological materials to study molecular changes associated with neurodegenerative processes. The buccal epithelium deserves special attention, as it offers significant advantages thanks to easy and safe noninvasive sampling and a broader spectrum of molecular markers detectable in it as compared to blood lymphocytes.

Promising areas for further research include prospective validation of the identified molecular pattern complexes, standardization of methodological approaches, and integration of molecular data with clinical, neuropsychological, and neuroimaging measures to gain a deeper understanding of the pathophysiology of dementia.

Comprehensive molecular profiling of peripheral tissues may contribute to the development of biologically based models of neurodegenerative diseases. Further development of methods and algorithms will enable the transition from descriptive neurology to truly personalized and preventive medicine.

## Figures and Tables

**Table 1:** Functional roles of key biomarkers involved in neurodegenerative processes of the central nervous system.

Biomarker	Functional Role in Neurodegenerative Processes
β-amyloid	Forms neurotoxic oligomers and fibrils that disrupt synaptic transmission, induce oxidative stress, activate microglia, and promote tau hyperphosphorylation. Leads to the formation of amyloid plaques—a key hallmark of AD
CD34 protein	Altered CD34 expression reflects endothelial dysfunction and impaired neurovascular remodeling; associated with blood–brain barrier (BBB) disruption and chronic inflammation.
Claudin-5	Loss of claudins compromises BBB integrity, allowing immune cells and plasma proteins to penetrate brain tissue, thereby enhancing neuroinflammation and neuronal injury.
DRP1 protein	Excessive activation of DRP1 causes pathological mitochondrial fragmentation, energy deficiency, calcium imbalance, and neuronal apoptosis.
Transcription factor NF-κB	Chronic activation of NF-κB in microglia and astrocytes maintains neuroinflammation, increases cytokine production and oxidative stress, and contributes to neuronal death.
PTEN-induced kinase 1 (PINK1)	Mutations in PINK1 impair mitophagy, leading to the accumulation of damaged mitochondria, elevated ROS levels, energy deficiency, and degeneration of dopaminergic neurons.
RAGE	Activation of RAGE enhances oxidative stress and inflammation, facilitates β-amyloid transport across the BBB, and promotes its accumulation in brain tissue.
S100 protein	At high extracellular concentrations, act as DAMP molecules by activating RAGE receptors, inducing glial activation, nitric oxide production, and neurotoxicity. Elevated S100B is associated with gliosis and BBB dysfunction.
α-synuclein	Conformational changes and aggregation of α-synuclein disrupt synaptic transmission, axonal transport, and mitochondrial function, and promote the spread of pathology between neurons.
Tau protein	Hyperphosphorylated tau dissociates from microtubules, forming neurofibrillary tangles that impair axonal transport and lead to neuronal death.
Endothelin-1	Elevated endothelin-1 levels cause cerebral vasoconstriction, hypoperfusion, oxidative stress, and BBB damage, contributing to cognitive decline and ischemic neuronal injury.

**Table 2:** Analytical methods for determining neurodegenerative biomarkers.

Method/Platform	Biomarker	Sample	LoD *	Advantages	Restrictions
Enzyme-linked immunosorbent assay (EIA)	Aβ42, total tau-protein (t-tau), phosphorilated tau-protein 181 (p-tau181)	Cerebrospinal fluid (CSF)	low pg/mL	Standard for clinical diagnosis of AD; availability	Invasive; poor suitability for screening
Immunochemiluminescent assay (ICLA)	Aβ42	Blood plasma (BP)	~1 pg/mL (0.25–500 pg/mL)	Higher sensitivity; suitable for large samples	Lower sensitivity compared to digital methods
Automatic ICLA	Aβ42, t-tau, p-tau 181	CSF, BP	~2.8 pg/mL (Aβ42); ~0.28 pg/mL (p-тay181)	High precision; automation	Requires special equipment
Digital immune analysis (Simoa^®^)	Aβ42/40, p-tau181, NfL	BP	Femtograms–sub-pg/mL	Ultra-sensitivity; high correlation with PET and CSF	High cost
Mass spectrometry methods (LC–MS/MS)	Aβ42/40	BP	~10–2500 pg/mL	High specificity; high correlation with PET	Requires complex training and equipment
Surface-enhanced Raman spectroscopy (SERS)	Aβ42/40, t-tau, p-tau181	BP	Sub pg/mL–femtomoli	Simultaneous detection of multiple markers	Technological complexity
Electrochemical sensor	p-тay181	BP	~1.9 fg/mL	Very low LoD	Requires further clinical validation
Optical label-free biosensor	Aβ42	BP	~12 фг/мл	High sensitivity; real-time operation	Requires special equipment
Exosome analysis + immunoassay	t-tau, p-tau, exosomes	BP, serum	Low pg/mL	High sensitivity	Lack of standardization

* LoD values are taken from the literature; exact values depend on the specific kit, platform, and laboratory.

**Table 3:** Distribution of the proportion of cells with biomarker expression in peripheral blood lymphocytes and buccal epithelium, %.

Biomarker	Cell Type	Alzheimer’s Disease (Me, Q1–Q3)	Vascular Dementia (Me, Q1–Q3)	Control Group (Me, Q1–Q3)
β-amyloid	Lymphocytes	5.8 (1.1–10.5)	4 (0–8.9)	3.1 (0–10)
	Buccal epithelium	41.4 (27.6–54.3) *	45.9 (31.3–60) *	60.7 (50–67.9)
CD34 protein	Lymphocytes	6.3 (2.1–10.4)	4.2 (0–15.9)	5.7 (0–7.7)
	Buccal epithelium	16 (11.1–22.6)	18.8 (9.8–23.1)	15.9 (10.3–28.8)
Claudin	Lymphocytes	4.9 (0–8.1)	9.1 (4–19.7)	8.5 (2.4–25)
	Buccal epithelium	45.9 (33.3–55.2)	44.2 (40–46.8) *	52.8 (46.9–57.1)
DRP1 protein	Lymphocytes	5.6 (2.7–8.2) *	5.9 (2.4–8.3) *	1.2 (0.9–2.2)
	Buccal epithelium	36.1 (15.4–46.9)	28.6 (17.9–50)	29.4 (18.2–46.2)
Transcription factor NF-kB	Lymphocytes	3.9 (0–7.2)	4.0 (1.2–8.2)	2.6 (0.8–10.3)
	Buccal epithelium	47.1 (33.3–69.6) *	54.3 (21.4–57.1) *	79.2 (61.5–81.3)
PTEN-induced kinase 1 (PINK1)	Lymphocytes	7.4 (3.3–13.2)	10.6 (7.7–15.4)	3.6 (0–7.8)
	Buccal epithelium	57.7 (25–84) *^,^**	85.6 (76.6–91.3)	86.1 (81.2–90.7)
RAGE protein	Lymphocytes	2.1 (0–2.8)	6.9 (2.6–15.8)	0.6 (0–2)
	Buccal epithelium	0.0 (0–14.3) *^,^**	64.2 (21.8–77.1)	41.4 (10–70.8)
S100 protein	Lymphocytes	11.7 (0–22.4) *	8 (0–18.8)	3.7 (0–12.1)
	Buccal epithelium	59.9 (45.7–78.3) *	72.8 (67.9–92.3)	84.2 (76.2–88)
α-synuclein	Lymphocytes	9.9 (4.7–16.7)	9.7 (5.5–16.9)	15.8 (4.9–25)
	Buccal epithelium	5.3 (0–13) *^,^**	32.1 (10.3–50)	31 (17.2–50)
Tau protein	Lymphocytes	6.9 (2.9–14.8)	7.5 (0.5–19.7)	8.7 (1.3–15.2)
	Buccal epithelium	58.8 (47.6–67.9) *	62.5 (45.5–71.4) *	69.6 (53.1–85.7)
Endothelin-1	Lymphocytes	7.2 (4.3–14.3)	5.8 (2.4–12.0)	5.8 (2.2–15.0)
	Buccal epithelium	29.6 (15.6–44.7)	37.5 (14.3–57.7)	35.4 (8.8–66.7)

Notes: * Significance level *p* < 0.05 as compared to the control group. ** Significance level *p* < 0.05 as compared to the VD and AD groups.

**Table 4:** A complex of molecular patterns associated with cognitive impairment.

Biomarker Localization	Peripheral Blood Lymphocytes		Buccal Epithelium				
Biomarker	DRP1	S100	β-Amyloid	NF-κB	Tau Protein	Claudin	S100
Reference values of biomarker expression rate in healthy controls (%)	1.2	3.7	60.7	52.8	69.6	52.8	84.2
Biomarker expression rate in patients with AD (%), average	8.3	28.2	32.2	48.4	65.5	44.3	67.8
Comparison of biomarker expression in patients with dementia with the reference value of healthy volunteers	↑	↑	↓	↓	↓	↓	↓

↑—biomarker expressions in patients with dementia are higher than in healthy volunteers; ↓—biomarker expressions in patients with dementia are lower than in healthy volunteers.

**Table 5:** Complex of molecular patterns associated with Alzheimer’s disease.

Biomarker Localization	Buccal Epithelium			
Biomarker	Synuclein	RAGE	PINK1	Tau Protein
Reference values of biomarker expression rate in patients with vascular dementia (%)	32.1	64.2	85.6	12.5
Biomarker expression rate in patients with AD (%), statistic average	5.3	11.1	57.7	58.8
Comparison of biomarker expression in AD patients with reference values for patients with vascular dementia	↓	↓	↓	↑

↑—biomarker expressions in patients with dementia are higher than in healthy volunteers; ↓—biomarker expressions in patients with dementia are lower than in healthy volunteers.

## Data Availability

No new data were created or analyzed in this study.
